# A weighted bag of visual words model for predicting fetal growth restriction at an early stage

**DOI:** 10.3389/fmed.2025.1529666

**Published:** 2025-06-17

**Authors:** Ani Dong, Yiheng Zhang, Weiling Li, Mengjie Chen

**Affiliations:** ^1^School of Artificial Intelligence, Dongguan City University, Dongguan, Guangdong, China; ^2^School of Computer Science and Technology, Dongguan University of Technology, Dongguan, China; ^3^Department of Medical Ultrasonics, The Eighth Affiliated Hospital of Sun Yat-sen University, Shenzhen, China

**Keywords:** placenta, fetal growth restriction, visual word bag model, weight scaling, broad learning

## Abstract

**Purpose:**

Fetal growth restriction (FGR) is a significant concern for clinicians and pregnant women, as it is associated with increased fetal and neonatal mortality and morbidity. Although ultrasound has been the gold standard for many years to define FGR, it remains less than ideal for early detection of FGR. Placental dysfunction is a key factor in the development of FGR. The objective of this study is to achieve the early detection of FGR through the utilization of placental ultrasound images.

**Methods:**

A retrospective analysis was conducted using 80 placental ultrasound images from 40 FGR fetuses and 40 normal fetuses matched for gestational age. Approximately 300 texture features were extracted from the placental images using key texture feature selection and histogram of oriented gradients (HOG) extraction methods. These features were then re-encoded using a bag-of-visual-words model with weight scaling, resulting in more effective features. The encoded image features were used to train a classifier, and ensemble prediction techniques were used to improve classification accuracy.

**Result:**

In this study, we applied the proposed method alongside several popular image classification methods for predicting FGR. The proposed method achieved the best experimental results, with an accuracy of 70% and an F1 score of 0.7653. We also compared different feature extraction methods separately, and the experimental results showed that HOG feature extraction is more suitable for feature extraction of ultrasound placental images. Finally, we plotted the receiver operating characteristic (ROC) curve with an area under the curve (AUC) value of 0.80.

**Conclusion:**

To enable early prediction of FGR, we propose a visual bag-of-words model based on weight scaling for analyzing placental ultrasound images in the early stages—before significant fetal impairment occurs. The proposed model shows strong potential to assist doctors in making preliminary assessments, thereby facilitating earlier intervention. This can help reduce the risk of harm to both fetuses and pregnant women.

## 1 Introduction

Fetal growth restriction (FGR) refers to the failure to achieve expected physiological development which may lead to fetal deformities even fetal death (1, 2). According to a previous study, the prevalence of FGR is on the rise, with a global incidence rate as high as 10% by 2024 (3). Early intervention strategies, such as medication administration during the initial stages of pregnancy, have been shown to mitigate fetal morbidity and reduce risks to maternal health. Therefore, it is particularly important to achieve early detection of FGR.

FGR can be caused by multiple factors, including maternal diseases, fetal abnormalities, and placental dysfunction. Research has increasingly identified placental dysfunction as a key factor in the development of FGR (4, 5). When there is dysfunction in placental function, pathological alterations may manifest. These alterations can be identified using diagnostic imaging modalities such as ultrasound or magnetic resonance imaging, facilitating subsequent investigation and evaluation (6, 7). It inspired us if we can learn pattern from placenta ultrasound and associate it with its corresponding fetal FGR results, then we may achieve early detection of FGR.

FGR detection with placental ultrasound image is essentially an image classification task. In recent years, a variety of image classification models have been developed, with a primary focus on utilizing neural networks (8–10). However, neural network models require a significant amount of training data, which is frequently limited in this domain.

It is worth noting that, following the direction of healthcare professionals, it has been observed that the distinction between FGR and placental images of healthy fetuses is contingent upon the varying repetition times of regions of interest (ROIs) exhibiting distinct textures. Specifically, placental images of FGR tend to show short rod-like textures, while placental images of normal fetuses typically have dot-like textures. To take advantage of this feature, we came up with the idea of using a visual word bag model.

However, ultrasound imaging exhibits reduced resolution. In the early stage, the structural distinctions between the placenta of fetuses with FGR and that of normal fetuses are slight, resulting in minimal variations in pattern within ultrasound images. When the texture difference between FGR and normal images is very small, existing BOVW models are difficult to accurately encode them. Moreover, the long gestational period results in a limited number of data samples. Hence, a method which is able to maximize learning patterns from a small number of samples and accurately associate them with corresponding FGR results is desired.

To address the above issues, this study proposes a weighted bag of visual word (WBOVW) model for detecting fetal growth restriction at an early stage with 2-fold ideas:

(a) encoding placental ultrasound with a novel weighted bag of visual word model;
(b) learning association knowledge of the encoded placental ultrasound image and its corresponding FGR result using ensemble classifiers.

The contribution of this study is as follows:

(a) achieving FGR detection at early pregnancy with only placental ultrasound images;
(b) providing a weighted bag of visual word method.

Experimental results on real ethically certified data collected by a hospital indicate that the proposed method can detect FGR at an early stage.

## 2 Materials and methods

### 2.1 Data

From January 2019 to November 2023, ultrasound images of placenta from normal fetuses and FGR fetuses at a gestational age (GA) range of 20–32 weeks were collected from The Eight Affiliated Hospital of Sun Yat-sen University. All scans were performed using GE Voluson E8 or E10 machines (GE Healthcare, Zipf, Austria), equipped with a GE RM6C volumetric probe (frequency: 4–8 MHz). The images were obtained by three sonographers, each with at least 6 years of fetal ultrasound examination experience. All patients signed the informed consent form for a fetal ultrasound examination. This retrospective study was approved by the Ethics Committee of The Eighth Affiliated Hospital of Sun Yat-sen University (2023-055-01).

**Figure 1:**
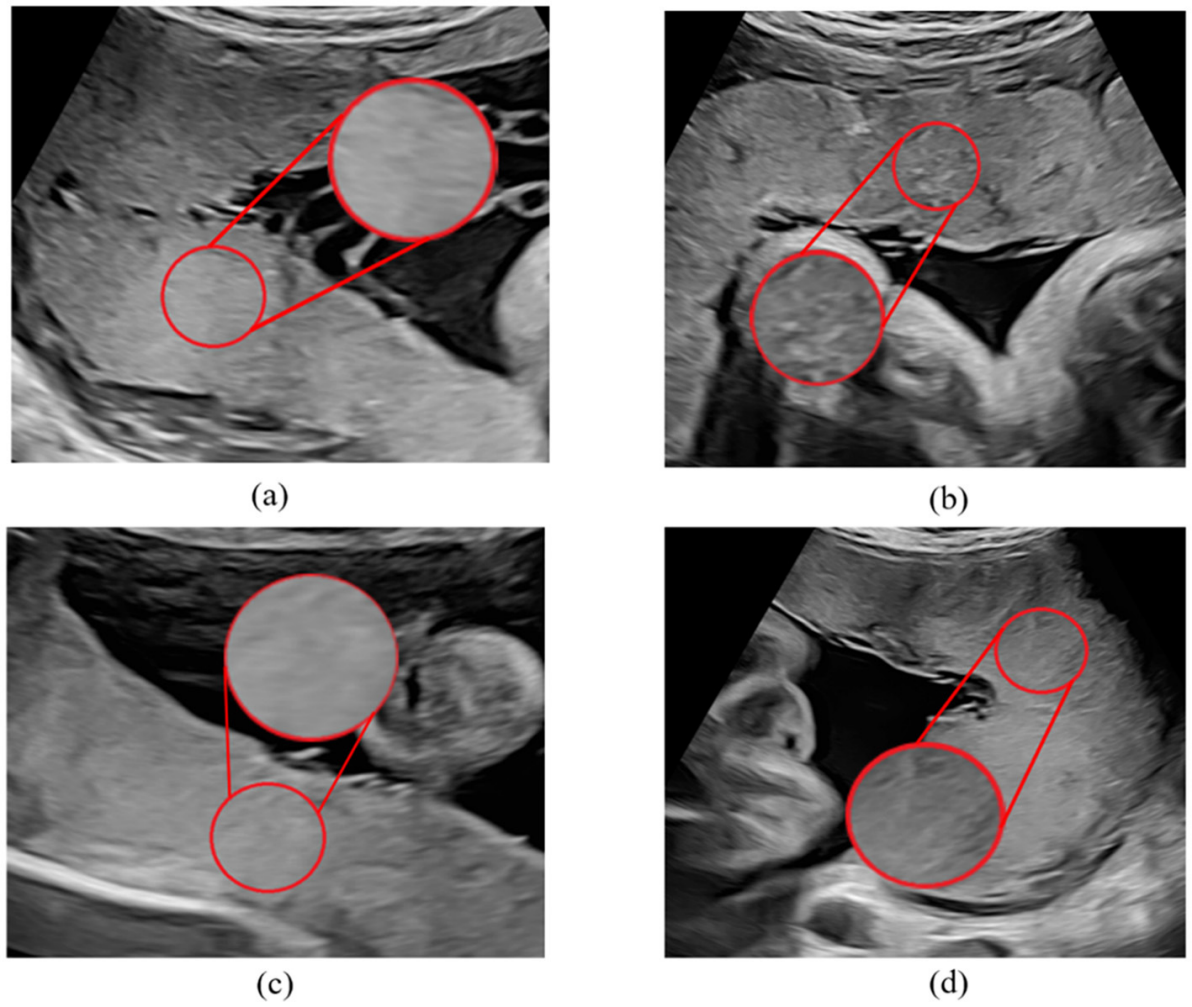
Sample of placental ultrasound images. **(a, c)** Are placental ultrasound images with FGR, and **(b, d)** are normal placental ultrasound images.

A total of 80 cases were enrolled in this study, including 40 normal fetuses and 40 FGR fetuses where the placentas were at maturity level 0-I. For FGR fetuses, we included the data before the first diagnosis of FGR on clinical. Exclusion criteria applied to 80 cases were structural fetal anomalies, multiple pregnancies, abnormal insertion of placenta umbilical cord, single umbilical artery, and fetal anomalies other than FGR. A sonographer with more than 6 years' experience of fetal ultrasound examination conformed ROI. The ROI was locked in a circular area with a diameter of 2 cm near the insertion point (Figure 1).

### 2.2 Framework

**Figure 2:**
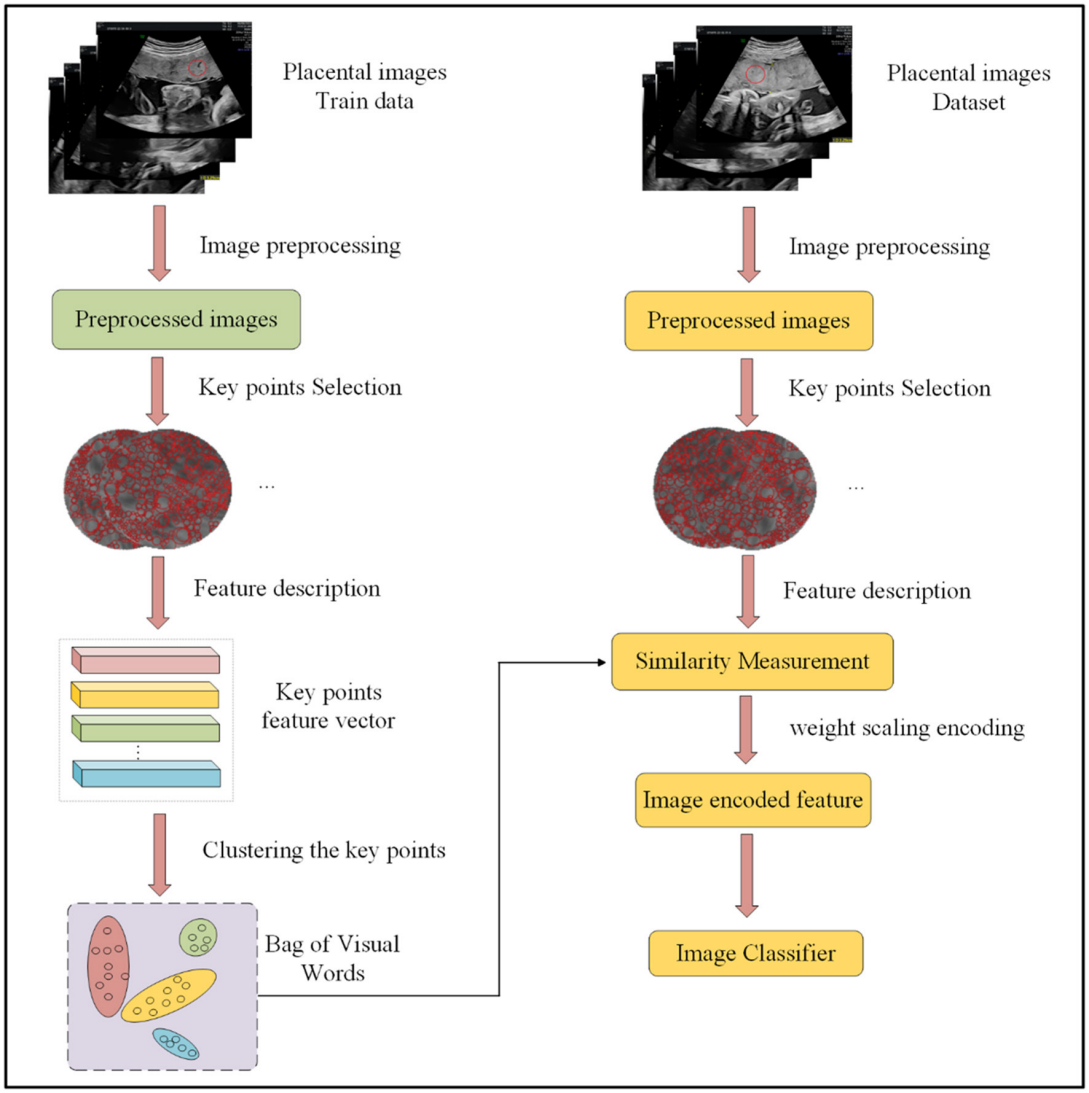
Overall framework of our method.

The proposed WBOVW model, illustrated in Figure 2, comprises two primary components: the establishment of a visual word bag library and image encoding classification. Construction of visual word bag library: We will use training set images to build this visual word bag library. Note that the input of WBOVW is placental ultrasound images. In each image, a ROI is marked by imaging physicians. The ROIs are segmented from images as the preprocessed image and feed to the key points feature extraction module. It selects key points from ROI with scale-invariant feature transform (SIFT) and learns features of each key point with histograms of oriented gradients (HOG). Afterward, the Gaussian mixture model (GMM) is used to construct visual words based on the extracted image block features of each ROI. Image encoding classification: This part will use a visual word bag library to feature encoding all images. Each image undergoes the same preprocessing and feature extraction stages as mentioned above. The extracted features of each image will be calculated for similarity with visual words and encoded with weight scaling. The encoded image features can be trained through a classifier, and finally, ensemble prediction is used to improve classification accuracy.

### 2.3 Key feature extract

Key point selection with SIFT key point detection: Scale-invariant feature transform (SIFT) is an efficient method for finding key points on different scale spaces (11). Notably, in placental ultrasound images, regions of interest (ROIs) designated by the data provider may vary in size. The precision of features extracted directly from the image is influenced by both the image size and the semantic arrangement of features. Therefore, we first employ SIFT key point detection to identify key points in each image.

The SIFT algorithm first applies a Gaussian filter to the image for filtering:


(1) $$\begin{array}{l} L\left(x,y,\sigma \right)=G\left(x,y,\sigma \right)⊗I\left(x,y\right), \end{array}$$

where *I*(*x, y*) is the input image, *G*(*x, y*, σ) is the Gaussian filter, ⊗ represents filtering operations, and *L*(*x, y*, σ) is the filtered image. The Gaussian filter is defined as follows:


(2) $$\begin{array}{l} G\left(x,y,\sigma \right)=\frac{1}{{2\pi \sigma }^{2}}e^{-\frac{{\left(x-m/2\right)}^{2}+{\left(y-n/2\right)}^{2}}{2\sigma^{2}}}, \end{array}$$

where σ represents the standard deviation of the Gaussian distribution, m and n denote the size of the Gaussian filter, and *x* and *y* represent the positions of the corresponding elements.

**Figure 3:**
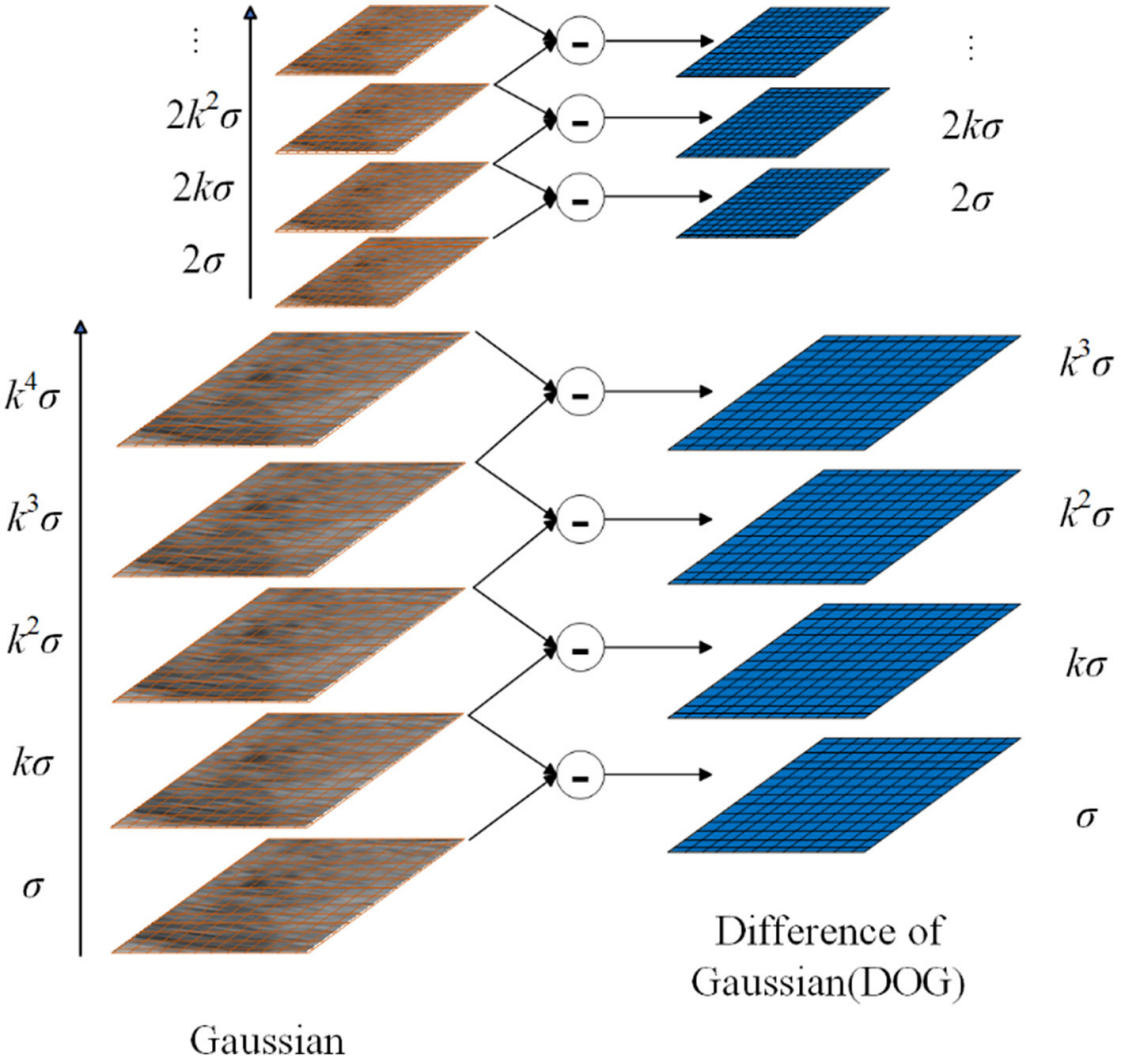
Overview of DOG.

To find the extreme points of the image, by subtracting the adjacent image matrices in the same scale space, the Gaussian difference scale space can be obtained as follows:


(3) $$\begin{array}{l} D\left(x,y,\sigma \right)=L\left(x,y,k\sigma \right)-L\left(x,y,\sigma \right), \end{array}$$

where *k* is the scale factor. The Difference of Gaussians (DoG) model is illustrated in Figure 3.

After establishing the DoG, we search for local extrema points. Each pixel is compared with its neighbors to check whether it is larger or smaller than all its neighbors. The adjacent point is composed of 8 adjacent points of the same scale and 9 × 2 points of adjacent scales up and down, a total of 26 points. This results in key points at different scales in the image, increasing diversity in BOVW construction.

#### 2.3.1 Key point feature extraction with HOG

The domains of different sizes around each key point were selected based on their scale size, and HOG feature extraction was performed on this domain (12). First, the domain was divided into 4 × 4 = 16 sub-regions and the pixel gradient size and direction were calculated in each sub-region.


(4) $$\begin{array}{l} G_{x}\left(x,y\right)=I\left(x+1,y\right)-I\left(x-1,y\right), \end{array}$$

(5) $$\begin{array}{l} G_{x}\left(x,y\right)=I\left(x,y+1\right)-I\left(x,y-1\right), \end{array}$$

(6) $$\begin{array}{l} G\left(x,y\right)=\sqrt{G_{x}^{2}+G_{y}^{2}}, \end{array}$$

(7) $$\begin{array}{l} \varphi \left(x,y\right)=\text{arctan }\frac{G_{y}\left(x,y\right)}{G_{x}\left(x,y\right)}, \end{array}$$

where *G* (*x, y*) denotes the gradient size representing pixels, and Φ (*x, y*) denotes the gradient direction of pixels.

Then, we divide the range of 0°-180° into nine intervals: (0°, 20°), (20°, 40°), , (160°, 180°). For each sub-region, the gradient size is accumulated and normalized based on the interval corresponding to the pixel gradient direction value. Finally, the feature descriptors of 16 sub-regions were concatenated together to obtain *V*_*i, j*_, representing the feature vector of key point *j* in image *i*.

### 2.4 Encoder

#### 2.4.1 Building the bag of visual words with Gaussian mixture model

Through key feature representation component, we obtained feature matrix *V*_*i*_*R*^*min*^ for *i*-th placenta ultrasound image, where *m*_*i*_ represents the number of key points in image *i*, and *n* represents the feature vector of the key points. With *V*_*i*_, the key feature of the *i*-th placenta ultrasound image can be represented. However, it cannot be utilized for prediction directly since (a) *V*_*i*_ has different size; and (b) the order of ROIs recorded by *V* brings additional spatial semantic information.

To fully utilize the key features, a method that can recode them is required. BOVW (13, 14) has proven to be effective in recoding features. The feature encoding technique employed by BOVW relies on statistical analysis which can eliminate the influence of semantic order. Traditional BOVW methods typically use k-means clustering to cluster the feature vectors. However, in this study, the clustering model employed is the GMM (15), which not only incorporates the distance information of the sample points but also includes information about the number of sample points and their variances. We use GMM to cluster the key points of all images in the training set to construct BOVW.

In GMM, it is assumed that the data are composed of several Gaussian distributions. The formula for the Gaussian mixture model is as follows:


(8) $$\begin{array}{l} \text{P}\left(V_{i,j}\right)={\sum_{k=1}^{K}\pi }_{k}N\left(V_{i,j}|u_{k},\Sigma_{k}\right), \end{array}$$

where *u*_*k*_denotes the mean factor of Gaussian distribution, ∑_*k*_ is the covariance, π_*k*_is the weight of each Gaussian model, and *K* is the number of Gaussian models.

Note that the goal of GMM is to maximize the likelihood function as follows:


(9) $$\begin{array}{l} L=\prod_{i=1}^{I}\prod_{j=1}^{Ji}P\left(V_{i,j}\right), \end{array}$$

where *I* is the number of images, and *J*_*i*_ is the number of key points in *i*-th image. Hence, these parameters in Equation 8, i.e., *u*_*i*_, ∑_*i*_, and π_*i*_, can be derived with Expectation-Maximization (EM) algorithm. By implementing the aforementioned process, a dictionary can be obtained for supporting image encoding.

#### 2.4.2 Encoding input placental ultrasound image

**Figure 4:**
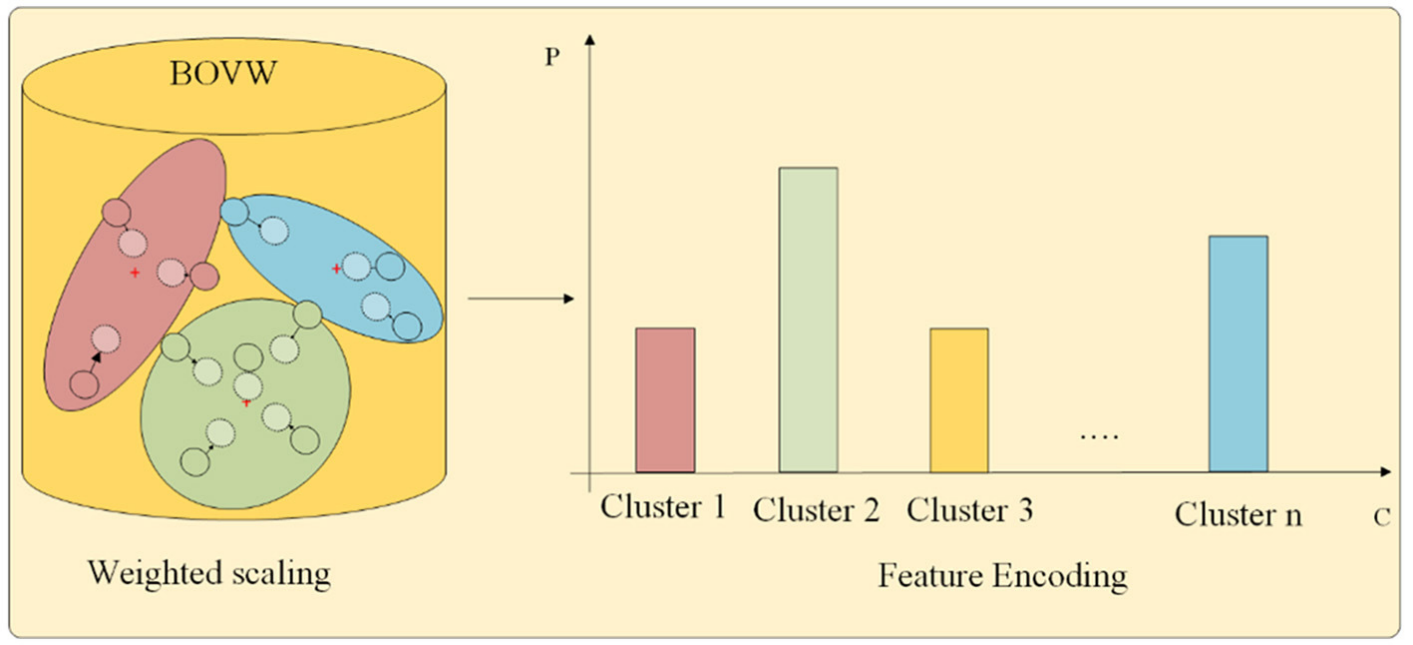
Feature encoding.

Recalling that we use the GMM to calculate the probability of key points belonging to each cluster, which includes distance information, covariance information, and weight information. Ideally, each key point should be assigned to a specific cluster, which means that the probability of belonging to a specific cluster class should be relatively high. However, due to the low resolution of medical images and the influence of noise, some key points may fall at the intersection of certain clusters. It belongs to different cluster classes with similar probabilities, and weighted statistics based on this method may not be conducive to classification. To reduce the impact of noise, we propose a weight scaling statistical method in this study. The feature encoding part is shown in Figure 4.

First, we use the trained GMM to perform probability prediction on each key point in the image.


(10) $$\begin{array}{l} p_{i,j,k}=\pi_{k}N\left(V_{i,j}|u_{k},\sum_{k}\right), \end{array}$$

where *p*_*i*_,*j, k* is the probability of *V*_*i, j*_ belonging to the *k*-th cluster.

The labels of each cluster are identified to which the key points belong based on probability values. Let *P*_*i, j*_ be a length *K* vector, e.g., *p*_*i, j*_*,1*, …, *p*_*i, j, k*_, …, *p*_*i, j, k*_,. A length *K* vector, e.g., *Q*_*i*_,*j*= (*q*_*i, j*_*,1*, …, *q*_*i, j, k*_, …, *q*_*i, j, k*_), is proposed to encode the *j*-th key point of *i*-th image, where *q*_*i, j, k*_ϵ{0, 1} and ∑*q*_*i, j, k*_ = 1. Moreover, *q*_*i, j, k*_ = 1 if *p*_*i, j, k*_ is the maximum among all *p*s. Then, the weight scaling formula is as follows:


(11) $$\begin{array}{l} U_{i}=\frac{\overset{J}{\underset{j=1}{\sum }}\alpha *P_{i,j}+\left(1-\alpha \right)*Q_{i,j}}{J}, \end{array}$$

where *U*_*i*_ represents the re-encoded features of image *i*, and α is a weight scaling hyperparameter.

The above approach can cause feature points to move toward the corresponding cluster center. There are significant differences in histogram statistics for key points located at certain cluster boundaries. When α = 1, it becomes a probability-weighted average, and when α = 0, it becomes a class-weighted statistic. Introducing the α hyperparameter allows for better adjustment, depending on different datasets and their specific requirements. After feature encoding, each image has a corresponding feature vector *U*_*i*_ with same dimension.

### 2.5 FGR predictor

**Figure 5:**
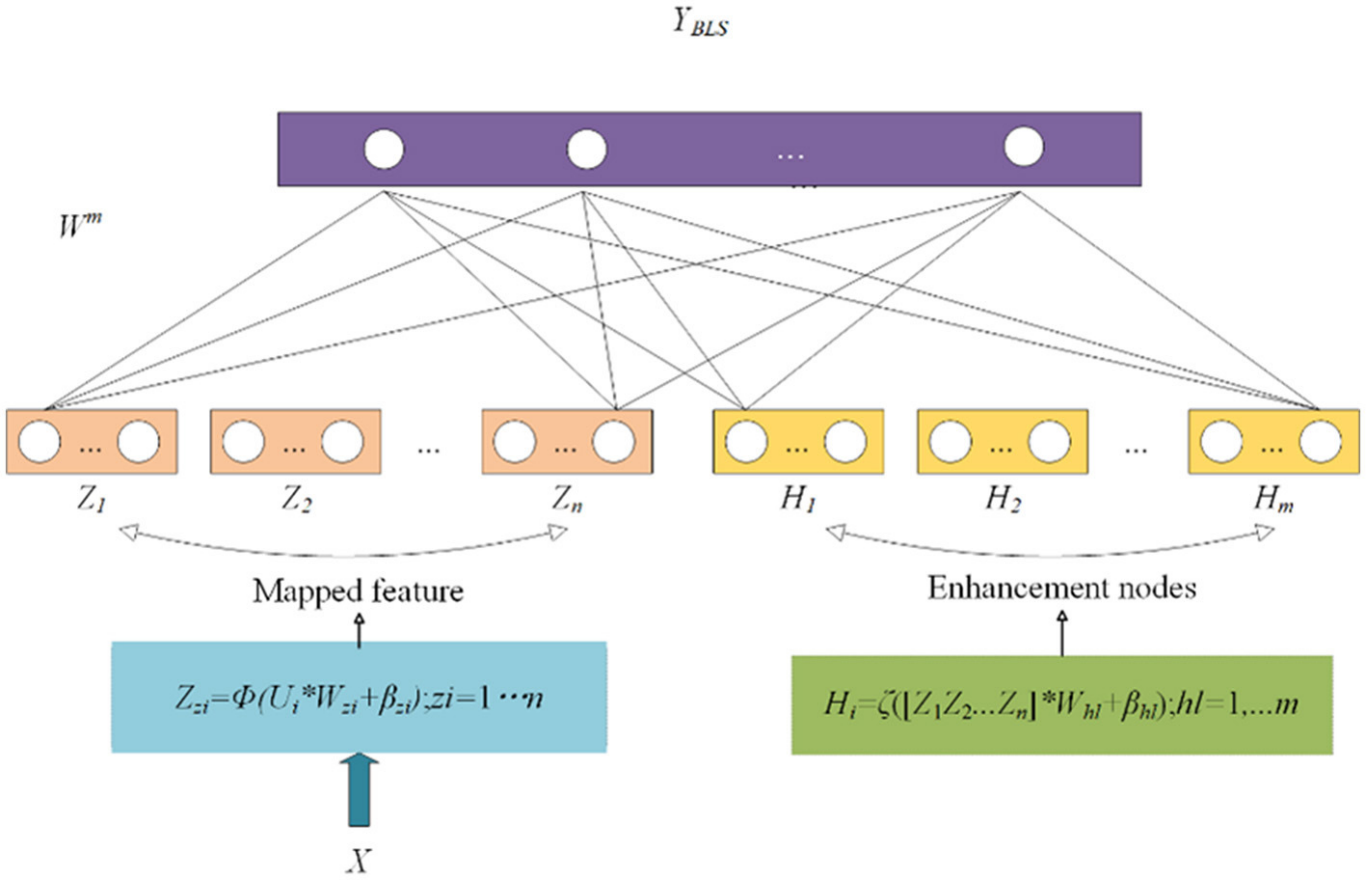
Structure of broad learning system.

Currently, many models use fully connected neural networks for classification. However, when the amount of data is small, there is a risk of overfitting. Therefore, it is not suitable for tasks such as FGR prediction. In machine learning, support vector machines (SVMs) use “kernel functions” to map data from the original feature space to a higher-dimensional feature space, making the data linearly separable in that space. It can be used to solve binary classification tasks. In addition, BLS involves mapping input features through non-linear transformations to a higher-dimensional feature space where data become more easily separable and classifiable (16, 17). It can be seen as a variant of fully connected neural networks, but while fully connected neural networks focus on constructing deep architectures, BLS focuses on constructing wide architectures, as shown in Figure 5.

BLS does not directly input the original feature vectors into the network. Instead, it first expands the original features through a feature mapping layer.


(12) $$\begin{array}{l} Z_{zi}=\phi \left({\text{U}}_{i}*W_{zi}+\beta_{zi}\right),zi=1....n, \end{array}$$

where *W*_*zi*_ and β_*zi*_ are parameters, Φ represents the non-linear transformation, and *Z*_*zi*_ represents the *l*-th node after the mapping node.

The nodes obtained in the previous step are then passed through an enhancement layer to obtain *H*_*l*_. Finally, *Z*_*l*_ and *H*_*l*_ are concatenated, and the concatenated result is passed through linear and non-linear transformations to output the predicted values.


(13) $$H_{hl}=\xi ([Z_{1}Z_{2}\;Z_{n}]*W_{hl}+\beta_{hl}),hl=1,.m,$$

where ζ represents the non-linear transformation, and *W*_*hl*_ and β_*hl*_ are parameters.


(14) $$\begin{array}{l} {\text{Y}}_{BLS}=\left[Z_{1},...Z_{n}|H_{1},...H_{m}\right]W^{m}=AW^{m}, \end{array}$$

where *W*^*m*^ represents the parameters, and Y_BLS_ represents the predicted probabilities from the BLS classifier.

The parameter *W*^*m*^ can be obtained through optimization and inverse matrix solution.


(15) $$\begin{array}{l} \underset{W^{m}}{\text{arg min}}:||AW^{m}-Y_{BLS}|{|}_{2}^{2}+\varepsilon ||W|{|}_{2}^{2}, \end{array}$$

(16) $$\begin{array}{l} W^{m}={\left(\varepsilon I+AA^{T}\right)}^{-1}A^{T}Y_{BLS}, \end{array}$$

where ε is the regularization parameter.

In broad learning, as the width increases, the risk of overfitting becomes more severe, and a larger amount of data is required. In fact, the accuracy of an ensemble of multiple smaller models may be higher than that of a single large model. BLS and SVM adopt different mapping methods to enhance data separability. In this study, we integrate these two classifiers for predictive modeling in medical tasks, aiming to improve the robustness of the model. The SVM classifier can record the distance of each sample to the hyperplane. We use the sigmoid function to convert this distance into a probability value. On the other hand, the last layer of the BLS outputs the score values for each class for each sample. We normalize the output values using softmax to convert them into probability values. Finally, we can use λ and 1-λ as weights to combine the probability values of the two models. The resulting combined probability is used for ensemble prediction. By adjusting the hyperparameter λ, we can control the mixing ratio of the two models.


(17) $$\begin{array}{l} {\text{Y}}_{\text{i,j}}=\frac{\exp\left(Y_{i,j}^{BLS}\right)*\lambda }{\sum_{j=0}^{1}\exp\left(Y_{i,j}^{BLS}\right)}+\frac{1*\left(1-\lambda \right)}{1+\exp\left(-dist\left(Y_{i,j}^{SVM},L\right)\right)}, \end{array}$$

In the formula, $Y_{i,j}^{BLS}$ represents the score assigned by the BLS classifier to sample *i* for class *j*, -dist ($Y_{i,j}^{SVM}$,L) represents the distance from the hyperplane when sample *i* is predicted as class *j* by SVM, L represents the constructed hyperplane by SVM, and *Y*_*i, j*_ represents probability of sample *i* belonging to class *j*.

## 3 Result

### 3.1 Experiment settings

#### 3.1.1 Implement details

**Figure 6:**
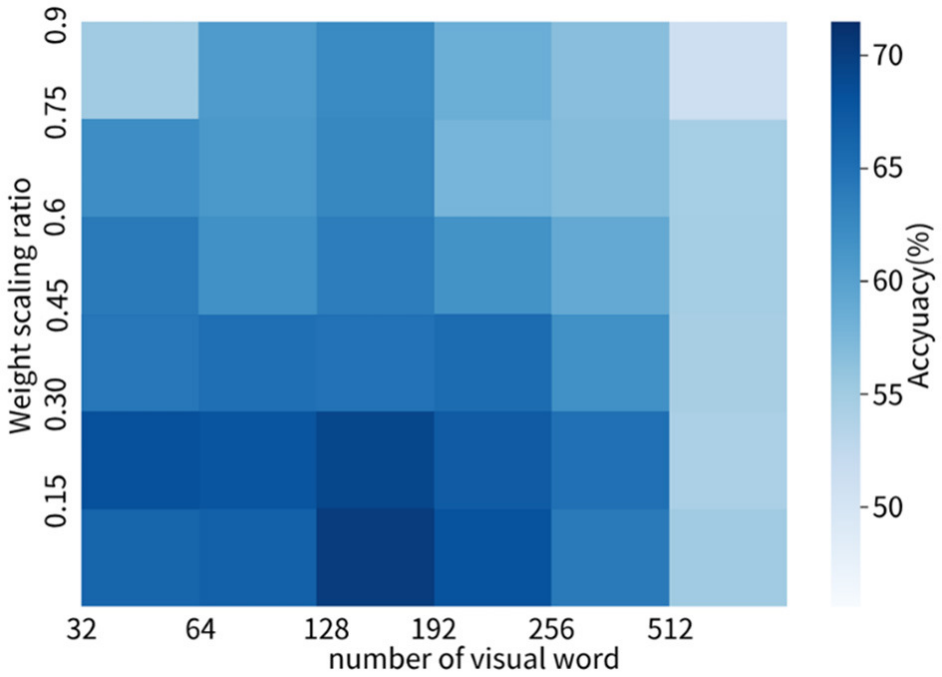
Heatmap of variation in the effect of different number of visual words and weight scaling ratio on model performance.

To evaluate the performance of the models, we used a cross-validation method, using a ratio of 6:2:2 for the training set to validation set to test set. This experiment has been repeated at least 10 times or more. All outcome indicators are obtained from the test set. In image preprocessing, wavelet transform is utilized for denoising. Due to the unstable position and angle of the ultrasound probe during image acquisition, as well as the small image dataset, we also adopt brightness variation, contrast transformation, random rotation, and random cropping as augmentation methods for each image. Note that we did not use operations such as random scaling and affine transformation as this would disrupt the original texture details of the placental image. To enhance the texture of the images, we used histogram equalization for texture enhancement. We obtained optimal model parameters through many experiments, as shown in Figure 6. We set the number of clusters in the bag-of-visual-words model to 128 (k = 128). In the weight scaling stage, we set α = 0.15.

#### 3.1.2 Feature extraction selection

**Figure 7:**
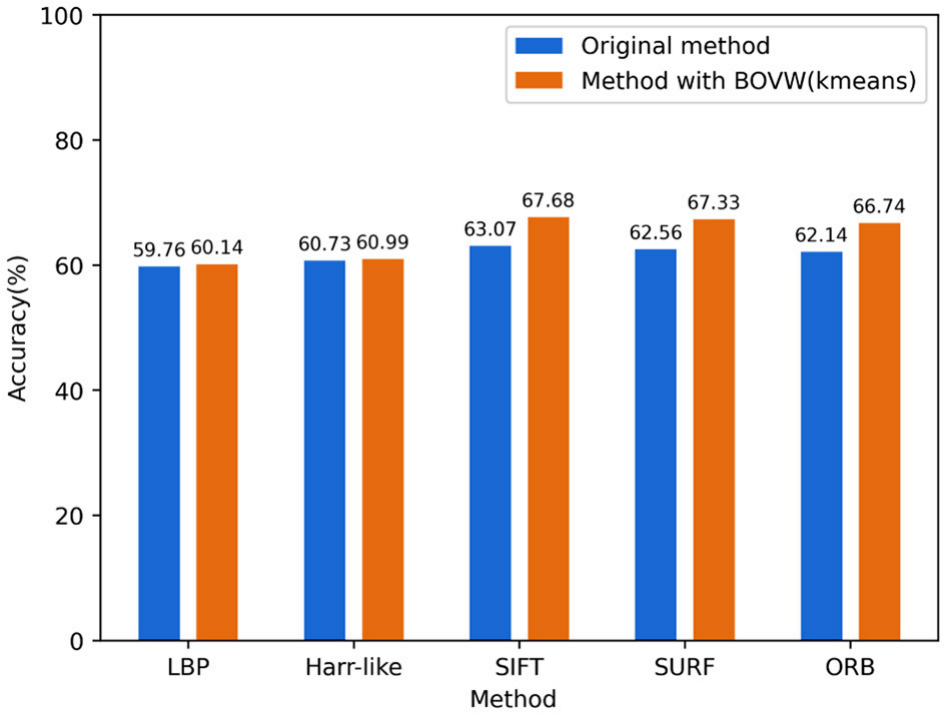
Comparison of feature extraction methods.

In terms of feature extraction methods, we selected mainstream image feature extraction methods for experiments, as shown in Figure 7. The accuracy of LBP and Harr-like feature extraction methods is relatively low, and the addition of the BOVW model for encoding does not improve the accuracy. This may be because the two methods mentioned above did not extract significantly different features and did not form clear clustering clusters, resulting in a smaller improvement in feature encoding. The SIFT feature extraction method has the best performance, with an accuracy of 63.07%. After BOVW feature encoding, the effect has been improved by 4.6%. SURF and ORB are improved algorithms of SIFT, which are much faster than SIFT but have lower accuracy.

### 3.2 Experiment results

#### 3.2.1 Ablation experiment

To understand the effectiveness of each component of the proposed model, we first conduct an ablation experiment. The experimental results are listed in Table 1. From this, we can conclude that each component has a positive contribution to the final prediction accuracy. For instance, with the addition of traditional BOVW feature encoding, the accuracy is 67.68%. After changing the clustering method to GMM clustering, the accuracy was significantly improved because GMM clustering can fit clusters of any shape. When we introduce our proposed GMM-scaled encoding, the accuracy is 70.68%, which is 1.35% higher than GMM.

**Table 1:** Results of the ablation experiment.

**SIFT**	**K-means**	**GMM**	**GMM-scaled**	**Accuracy**
√				63.07%
√	√			67.68%
√		√		69.33%
√			√	70.68%

**Figure 8:**
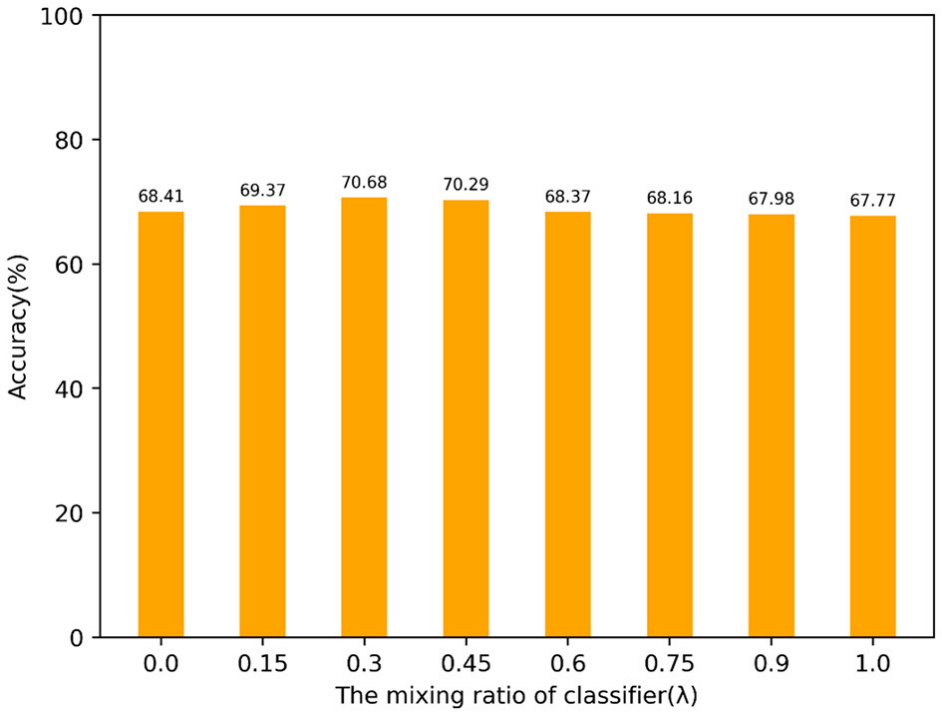
Mixing ratio of classifier.

Figure 8 shows our experiment on selecting the mixing ratio of the classifier. The models all selected SIFT and GMM scaled as feature extraction and encoding. The highest accuracy is achieved when the mixing ratio is λ = 0.3. When the performance of each model is good, model integration often brings a certain degree of accuracy improvement. We chose SVM and BLS as integrated models because their principles are similar, both of which remap features through non-linear transformations.

#### 3.2.2 Comparison against state-of-the-art models

The involved models are listed in Table 2. The comparative models, i.e., M1-M6, for the proposed model, i.e., M7, are deep learning-based models or BOVW variations. The experimental results are listed in Table 3; from this, we have the following findings:

**Table 2:** Involved models.

**No**.	**Description**
M1	GhostNet (18)
M2	ShuffleNetV2 (19)
M3	MobileNetV3 (20)
M4	BOVW model with distance and angle information (13)
M5	The improving bag-of-visual-words model via combining deep features with feature difference vector (21)
M6	The bag of visual words using neural network classifier for detection of COVID-19 in X-ray (14)
M7	The FGR prediction model proposed in this study.

**Table 3:** Comparison of experimental results.

**Model**	**Accuracy**	**Recall**	**Precision**	**F1-Score**
M1	65.56%	69.45%	76.71%	0.7289
M2	65.83%	69.97%	78.43%	0.736
M3	66.10%	70.20%	79.30%	0.7448
M4	65.27%	69.45%	76.71%	0.7290
M5	68.05%	71.33%	79.30%	0.7510
M6	67.78%	71.33%	77.58%	0.7432
M7	**70.68%**	**73.21%**	**80.17%**	**0.7653**

M7 outperforms all its peers on all evaluation metrics.

To conduct a comprehensive comparison, a number of widely utilized lightweight image classification models, denoted as M1–M3, were chosen for evaluation. In medical scenarios characterized by limited sample sizes, the utilization of lightweight models possessing fewer parameters can help mitigate issues related to overfitting. Notably, among these models, M3 (MobileNetV3) demonstrated the highest accuracy but only 66.10%. This finding implies that neural network models may encounter challenges in effectively extracting relevant features from ultrasound placental images.

To validate the proposed bag-of-visual-words (BOVW) approach, we chose to employ various iterations of the BOVW model, including M4–M6. Notably, the M5 variant demonstrated superior performance, achieving an accuracy rate of 68.05%, which signifies an improvement of 1.95% compared to M3. These results indicate the effectiveness of the BOVW model in feature extraction from placental ultrasound images. In addition, our model M7 yielded the most favorable results, with an accuracy of 70.68% and an F1 score of 0.7653.

#### 3.2.3 Confusion matrix and ROC

**Figure 9:**
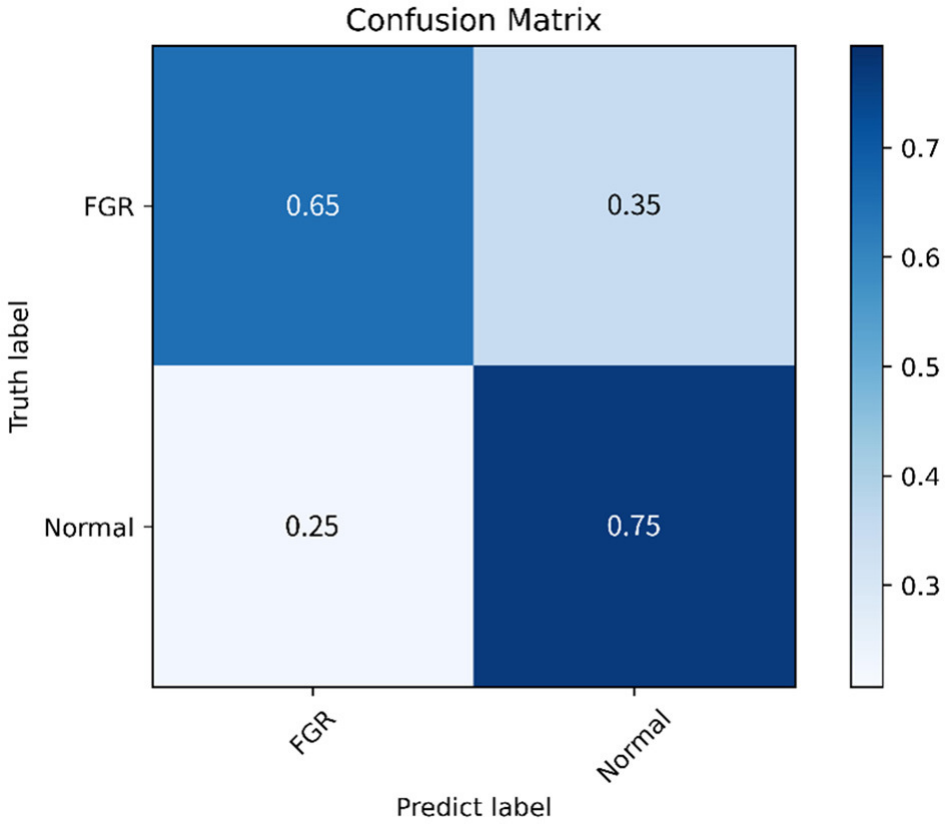
Confusion matrix.

**Figure 10:**
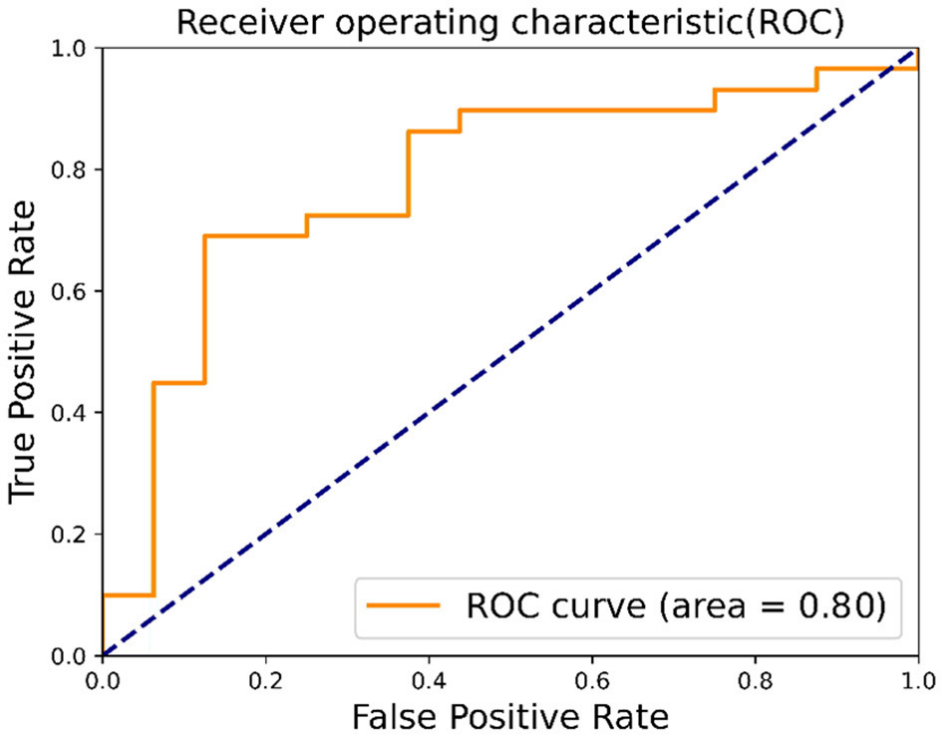
ROC.

This study further evaluates the clinical trial value of the proposed algorithm and draws the confusion matrix and receiver operating characteristic (ROC) curve. The confusion matrix, as depicted in Figure 9, illustrates the classification accuracy of each category, with the diagonal representing the accuracy. Figure 10 displays the ROC curve, a commonly utilized tool for assessing classifier performance and effectively distinguishing between positive and negative classes. In Figure 10, it exhibits an area under the curve (AUC) value of 0.80.

## 4 Discussion

From the experiment results, we have the following understanding of the proposed method:

(1) Detecting FGR through placental ultrasound images is essentially an image classification task. However, the noise caused by ultrasound leads to inaccurate representation. The experiment shows that our proposed encoding mode weakens the impact of noise and improves classification accuracy.
(2) This model for detecting FGR through images has the feasibility of clinical application. Assisting doctors in auxiliary diagnosis can reduce their workload. In some areas where medical equipment is underdeveloped, this application has a higher value.
(3) Obtaining cross-sectional images from ultrasound video streams and delineating ROI in the images requires professional expertise. We can find a method in subsequent work to assist doctors in delineating ROI and searching for cross-sectional areas. In addition, we can also combine placental image information and other biometric information for multimodal detection of FGR.

## 5 Contributions

To predict fetal growth restriction at an early stage, we propose a visual bag-of-words model based on weight scaling for analyzing placental ultrasound images of pregnant women in the early stages of pregnancy. Specifically, we introduce weight scaling during the feature encoding stage and design an ensemble classifier for FGR prediction. Through ablation experiments and comparative analysis, we validate the effectiveness of our approach, achieving an accuracy of 70% in predicting FGR using limited samples. The proposed model shows its potential to assist doctors in making preliminary assessments during early pregnancy, which greatly helps with treatment and allows patients to receive treatment as soon as possible, reducing the harm to pregnant women.

## Data Availability

The raw data supporting the conclusions of this article will be made available by the authors, without undue reservation.
